# Using 16S rDNA and metagenomic sequencing technology to analyze the fecal microbiome of children with avoidant/restrictive food intake disorder

**DOI:** 10.1038/s41598-023-47760-y

**Published:** 2023-11-20

**Authors:** Qina Ye, Shaodan Sun, Jian Deng, Xiaogang Chen, Jing Zhang, Suihua Lin, Hongxuan Du, Jinxiong Gao, Xiaoyin Zou, Xiaoling Lin, Yawen Cai, Zhuoming Lu

**Affiliations:** 1 Department of Traditional Chinese Medicine, Guangzhou Women and Children Medical Center, No. 9 Jinsui Road, Guangzhou, 510623 China; 2 Guangzhou University of Chinese Medicine, Guangzhou, 510405 China; 3 Department of Pediatrics, The First Affiliated Hospital of Guangzhou University of Chinese Medicine, Guangzhou, 510405 China

**Keywords:** Microbiology, Gastroenterology

## Abstract

To investigate the gut microbiota distribution and its functions in children with avoidant/restrictive food intake disorder (ARFID). A total of 135 children were enrolled in the study, including 102 children with ARFID and 33 healthy children. Fecal samples were analyzed to explore differences in gut microbiota composition and diversity and functional differences between the ARFID and healthy control (HC) groups via 16S rDNA and metagenomic sequencing. The gut microbiota composition and diversity in children with ARFID were different from those in heathy children, but there is no difference in the composition and diversity of gut microbiota between children at the age of 3–6 and 7–12 with ARFID. At the phylum level, the most abundant microbes in the two groups identified by 16S rDNA and metagenomic sequencing were the same. At the genus level, the abundance of Bacteroides was higher in the ARFID group (*P* > 0.05); however, different from the result of 16SrDNA sequencing, metagenomic sequencing showed that the abundance of Bacteroides in the ARFID group was significantly higher than that in the HC group (*P* = 0.041). At the species level, *Escherichia coli, Streptococcus thermophilus* and *Lachnospira eligens* were the most abundant taxa in the ARFID group, and *Prevotella copri*, *Bifidobacterium pseudocatenulatum*, and *Ruminococcus gnavus* were the top three microbial taxa in the HC group; there were no statistically significant differences between the abundance of these microbial taxa in the two groups. LefSe analysis indicated a greater abundance of the order *Enterobacterales* and its corresponding family *Enterobacteriaceae,* the family *Bacteroidaceae* and corresponding genus *Bacteroides,* the species *Bacteroides vulgatus* in ARFID group, while the abundance of the phylum *Actinobacteriota* and its corresponding class *Actinobacteria ,* the order *Bifidobacteriales* and corresponding family *Bifidobacteriaceae*, the genus *Bifidobacterium* were enriched in the HC group. There were no statistically significant differences in the Chao1, Shannon and Simpson indices between the Y1 and Y2 groups (*P* = 0.1, *P* = 0.06, *P* = 0.06). At the phylum level, Bacillota, Bacteroidota, Proteobacteria and Actinobacteriota were the most abundant taxa in both groups, but there were no statistically significant differences among the abundance of these bacteria (*P* = 0.958, *P* = 0.456, *P* = 0.473, *P* = 0.065). At the genus level, Faecalibacterium was more abundant in the Y2 group than in the Y1 group, and the difference was statistically significant (*P* = 0.037). The KEGG annotation results showed no significant difference in gut microbiota function between children with ARFID and healthy children; however, GT26 was significantly enriched in children with ARFID based on the CAZy database. The most abundant antibiotic resistance genes in the ARFID group were the vanT, tetQ, adeF, ermF genes, and the abundance of macrolide resistance genes in the ARFID group was significantly higher than that in the HC group (*P* = 0.041). Compared with healthy children, children with ARFID have a different distribution of the gut microbiota and functional genes. This indicates that the gut microbiome might play an important role in the pathogenesis of ARFID.

**Clinical trial registration**: ChiCTR2300074759

## Introduction

Feeding and eating difficulties (FEDs) include avoidant/restrictive food intake disorder (ARFID), anorexia nervosa (AN), rumination disorder (RD), binge eating disorder (BED) and bulimia nervosa (BN)^1^. In the 5th revision of the American Psychiatric Association’s Diagnostic and Statistical Manual of Mental Disorders (DSM-5)^2^, ARFID is characterized by low interest in food and eating, underweight and/or nutrition deficiency^3^, which leads to one or more of the following: significant weight loss (or failure to meet expected weight and height trajectories in children and adolescents); (2) nutritional deficiencies (such as iron deficiency anemia); (3) a dependence on nutritional supplements (i.e., oral or enteral formulas) to meet energy requirements without an underlying condition necessitating this; and (4) and/or significant interference with day-to-day functioning due to the inability to eat appropriately. Although there are no diagnostic age restrictions in ARFID, it is more commonly present in childhood and adolescence^4^.

The intestinal microecosystem is composed of trillions of microorganisms that maintain a dynamic physiological balance and promote host immunity, metabolism, energy balance, and neural development^5, 6^. The shaping and multiplication of the gut microbiome start at birth, and the modification of its composition depends on various factors, such as the atmosphere, genetics, diet and lifestyle^7^. The gut microbiota plays a vital role in digestion^8^ and correlates the gastrointestinal tract and the central nervous system by the gut–brain–microbiota axis^9^. Current studies show that the gut microbiota plays an important role in eating disorders^10–14^, however, most researches focus on anorexia nervosa^15^. Given the multitude of aspects, complexity and the limited data in ARFID, a multidisciplinary approach seems to be the best option, and researching the characteristics of gut microbes in children with ARFID, may help us obtain a better understanding and provide more help for the treatment of ARFID in the future.

## Materials and methods

### Patients

Between August 2022 and September 2022, 135 children with ARFID and healthy children from Guangzhou Women and Children Medical Center were recruited through posters and were divided into an ARFID group (ARFID, n = 102) and a healthy control group (HC, n = 33). Each participant was evaluated by our pediatricians who trained in diagnosing ARFID, and the ARFID module of the Eating Disorder Examination (child version)^16^ was used for the structured clinical interview.

### Inclusion criteria

(1) Patients were 3–12 years old; (2) Patients were diagnosed with ARFID; and (3) the disease course was ≥ 3 months.

### Exclusion criteria

(1) age < 3 years or > 12 years; (2) treatment with antibiotics, intestinal microbial preparations, or other immunological preparations in the previous month; (3) weight loss or growth retardation due to certain chronic underlying diseases such as chronic heart and lung disease, liver disease, rheumatic disease, kidney disease, or immunodeficiency; and (4) noncooperation with sampling regimens or parental refusal to participate.

### Specimen collection

Fresh fecal samples were collected into 10 ml stool containers (Batch No: GC-1022, Bioland) and were immediately frozen and stored at − 80 °C until analysis.

### DNA extraction and 16S rDNA amplicon sequencing

The SDS method was used to extract the total genomic DNA from the samples. DNA concentration and purity were monitored on 1% agarose gels. According to the concentration, DNA was diluted to 1 ng/μL with sterile water. The V3–V4 region of bacterial 16S rDNA was then amplified using the primers F: CCTAYGGGRBGCASCAG and R: GGACTACNNGGGTATCTAAT (Novogene Co., Ltd. Beijing, China). All PCR mixtures contained 15 μL of Phusion^®^ High-Fidelity PCR Master Mix (New England Biolabs), each primer at 0.2 μM and 10 ng target DNA, and cycling conditions consisted of a first denaturation step at 98 °C for 1 min, followed by 30 cycles at 98 °C (10 s), 50 °C (30 s) and 72 °C (30 s) and a final 5 min extension at 72 °C. An equal volume of 1X loading buffer (containing SYBR Green) was mixed with the PCR products, and electrophoresis was performed on a 2% agarose gel for DNA detection. The PCR products were mixed in equal proportions, and then a Qiagen Gel Extraction Kit (Qiagen, Germany) was used to purify the mixed PCR products. Following the manufacturer’s recommendations, sequencing libraries were generated with the NEBNext^®^ UltraTM IIDNA Library Prep Kit (Cat No. E7645). The library quality was evaluated on a Qubit@ 2.0 Fluorometer (Thermo Scientific) and Agilent Bioanalyzer 2100 system. Finally, the library was sequenced on an Illumina NovaSeq6000 platform, and 250 bp paired-end reads were generated.

### Metagenome sequencing

We selected a subset of specimens after MicroPITA analysis for metagenome sequencing. In brief, a Covaris ultrasonic fragmentation instrument was used to randomly interrupt the 350 bp fragment, and the whole library was prepared by terminal repair, adding an A tail, adding a sequencing joint, purification and PCR amplification. After the completion of library construction, Qubit2.0 was used for preliminary quantification, diluting the library to 2 ng/µl, and then an Agilent 2100 was used to detect the insert size of the library. After the insert size met the expectation, q-PCR was used to accurately quantify the effective concentration of the library (effective concentration > 3 nM) to ensure the quality of the library. After qualified library inspection, Illumina PE150 sequencing was carried out by pooling different libraries according to the requirements of effective concentration and target on-machine data volume.

### Bioinformatics and statistical data analyses

#### 16S rDNA

Paired-end reads were assigned to samples based on their unique barcodes and were truncated by cutting off the barcodes and primer sequences. FLASH (Version 1.2.11) was used to merge paired-end reads^17^. Quality filtering of the raw tags was performed using fastp (Version 0.20.0) software to obtain high-quality clean tags. The clean tags were compared with the Silva database, using Vsearch (Version 2.15.0) to detect the chimera sequences, and then the chimera sequences were removed to obtain the effective tags^18^. For the effective tags obtained previously, denoising was performed with DADA2 in QIIME2 software (Version QIIME2-202006) to obtain initial amplicon sequence variants (ASVs), and then ASVs with abundances less than 5 were filtered out^19^. Alpha diversity was calculated from 4 indices in QIIME2, including the Observed_otus, Chao1, Shannon and Simpson indices. Beta diversity was calculated based on unweighted UniFrac distances in QIIME2. LEfSe software (Version 1.0) was used to perform LEfSe analysis.

#### Metagenome

Readfq was used for preprocessing raw data from the Illumina sequencing platform to obtain clean data for subsequent analysis. Clean data need to be BLASTed to the host database to filter out reads that may come from host origin^20, 21^. MEGAHIT software (v1.0.4-beta) was used for assembly analysis of clean data, and scaftigs without Ns were obtained by breaking the resulting scaffolds from the N junction^22–24^. MetaGeneMark (V3.05) was used to perform ORF prediction for scaftigs (≥ 500 bp) of each sample, and entries with a length less than 100 nt in the prediction results were filtered out. For the ORF prediction results, CD-HIT software (V4.5.8) was used to eliminate redundancy and obtain the nonredundant initial gene catalog. Clean data of each sample were aligned to the initial gene catalog by using Bowtie2 (Bowtie2.2.4) to calculate the number of reads of the genes on each sample alignment. Based on the abundance of each gene in the gene catalog in each sample, basic information statistics were obtained. The gene sequences were compared with the KEGG, CAZy and CARD functional databases to obtain functional information.

### Statistical analysis

SPSS26.0 statistical software was used for data analysis. Measurement data with a normal distribution were represented by ($\overline{x}\pm s$), and a t test was used. Measurement data with a nonnormal distribution were represented by M (P_25_, P_75_), and the Mann‒Whitney U test was used. For all comparisons, the level of significance was set at 0.05.

### Ethics approval

This study was performed in line with the principles of the Declaration of Helsinki. This study was approved by the Ethics Committee of the Guangzhou Women and Children Medical Center, (date 15/03/2021/No.201B01).

### Consent to participate

Consent to participate Informed consent was obtained from all parents of the children.

## Result

### Patient demographics

Among the children, 102 were diagnosed with ARFID, and 33 were healthy. There were 50 males and 52 females in the ARFID group and 15 males and 18 females in the healthy control group (HC group). There was no significant difference in age (*P* = 0.352) or sex composition (*P* = 0.512). The main characteristics are summarized in Table 1.

**Table 1:** Patient demographics.

Parameter	Number	Age (months)	Male/Female	Weight (kg)	Height (cm)
ARFID	102	66 (53, 82)	50/52	15.9 (14.0, 19.0)	107.0 (100.0, 116.0)
HC	33	60 (48, 72)	15/18	20.0 (16.0, 22.5)	115.0 (106.5, 119.5)
*P*		0.352	0.512	< 0.001	0.011

### 16S rDNA sequencing results

After high-throughput sequencing of the fecal samples, we generated 2,676,787,184 valid sequences in total. The average effective length was 415.95 bp, and the average number of sequences was 24,785,066.52.

### Composition and diversity of the microbiome in the ARFID group and HC group determined by 16S rDNA sequencing

**Figure 1:**
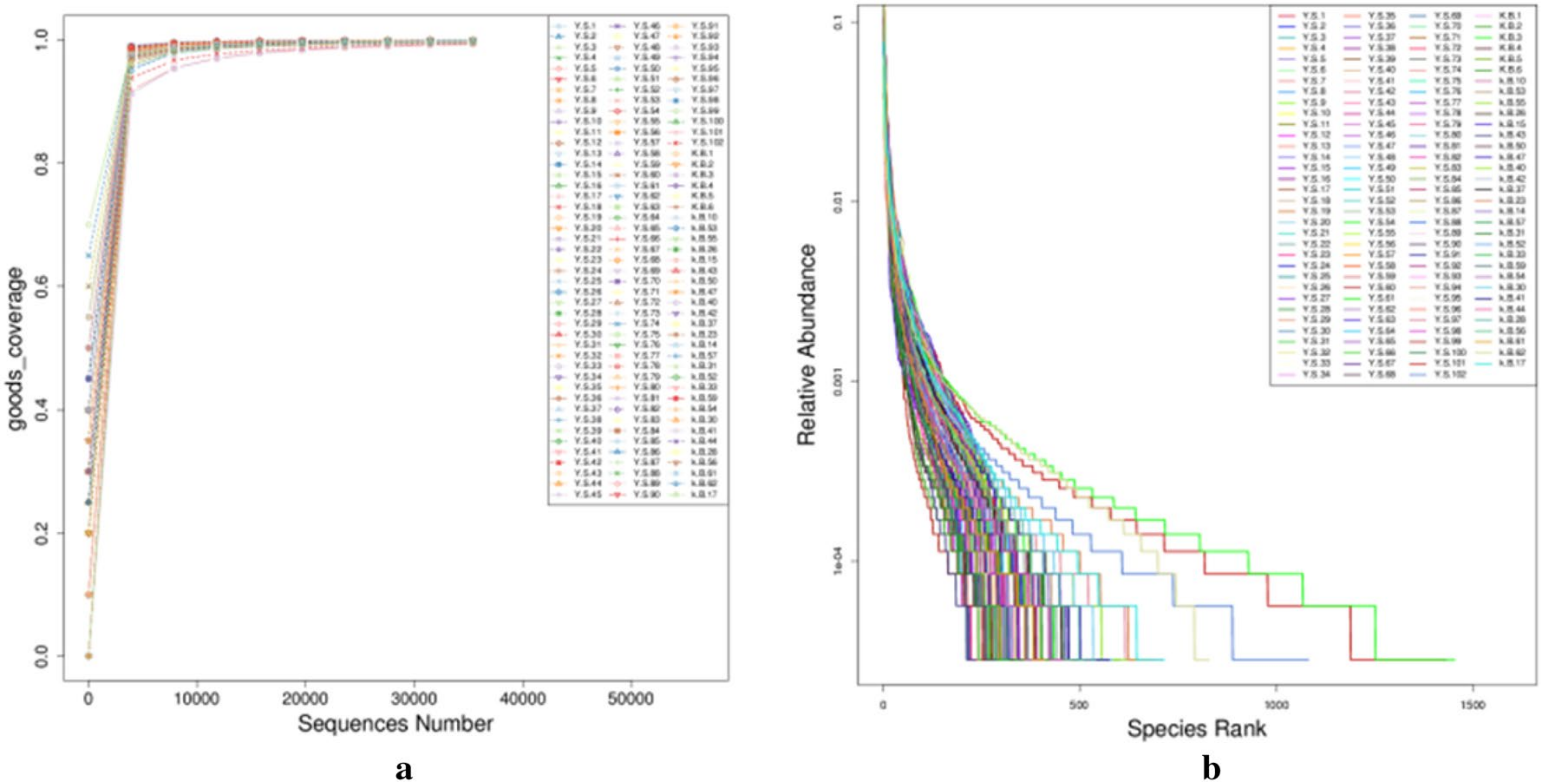
Microbiome composition of the fecal samples. Samples Y.S.1–Y.S.102 were in the ARFID group, and samples K.B.1–K.17 were in the HC group.

As shown in Fig. 1, the Goods coverage tended to be relatively stable, and the curve for the rank showed a downward trend with gentle leveling, which proves that the number of samples in this study was basically sufficient, and the sequencing depth could well reflect the complete microbiome composition of fecal samples from children with ARFID.

**Figure 2:**
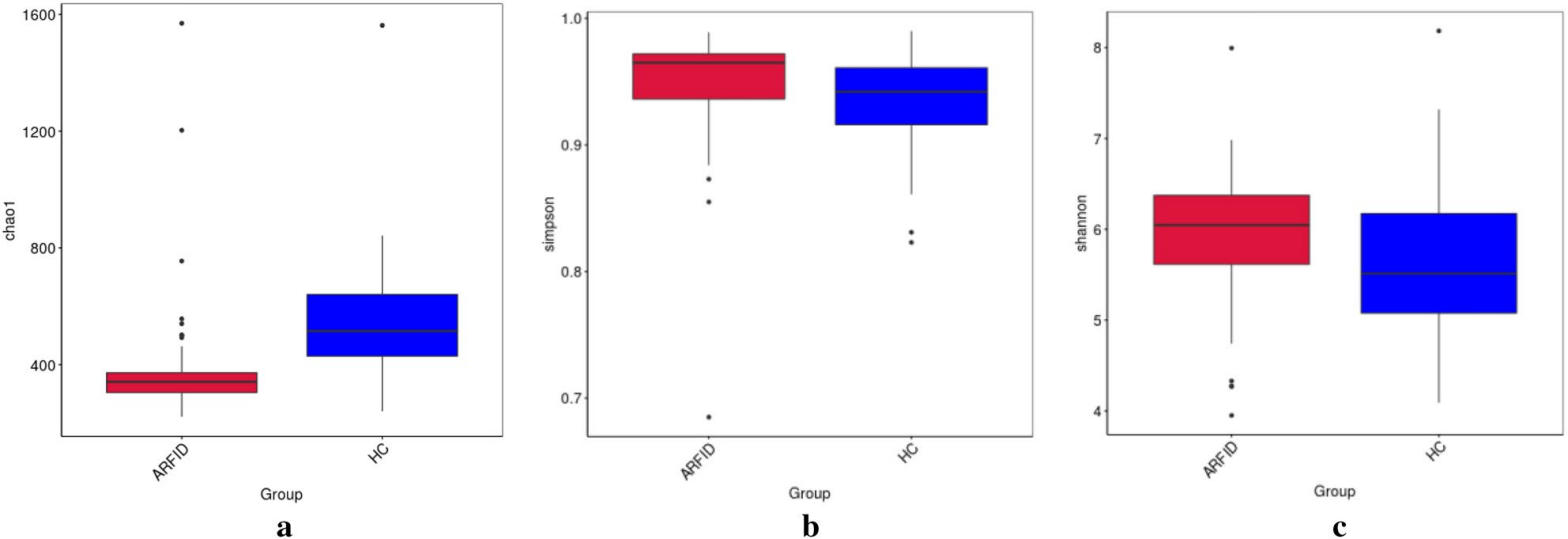
Comparison of α-diversity between the ARFID group and the HC group. (**a**) The Chao1 index values of the HC group were higher than those of the ARFID group; (**b**) the Simpson index values of the ARFID group were higher than those of the HC group; (**c**) the Shannon index values were significantly higher in the ARFID group than in the HC group.

The Chao1 index values of the HC group were higher than those of the ARFID group, and the differences were statistically significant (*P* < 0.001) (Fig. 2a). Additionally, the Simpson index values (Fig. 2b) and the Shannon index values (Fig. 2c) of the ARFID group were higher than those of the HC group, and the differences were statistically significant (*P* = 0.009 and *P* = 0.02).

**Figure 3:**
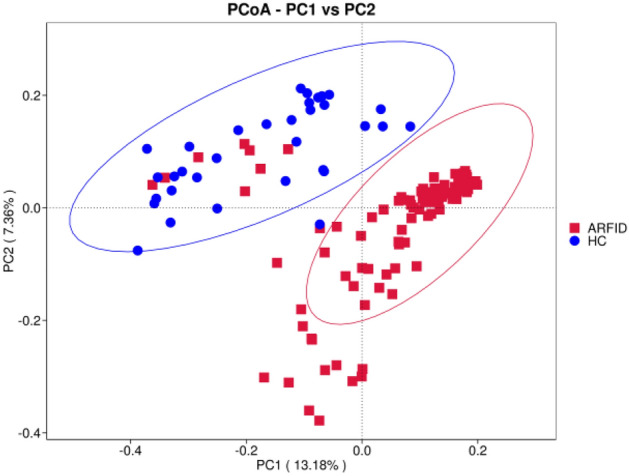
Each point in the figure represents a sample, and the points with the same color come from the same group. The closer the two points are, the smaller the difference in community composition is. Principal coordinate analysis (PCoA) plots of individual fecal microbiotas based on unweighted UniFrac distances.

We found two distinct groups of microbes using unweighted UniFrac distance-based principal coordinates analysis (PCoA), which showed that the microbiome of the ARFID group was different from that of the HC group (Fig. 3).

**Figure 4:**
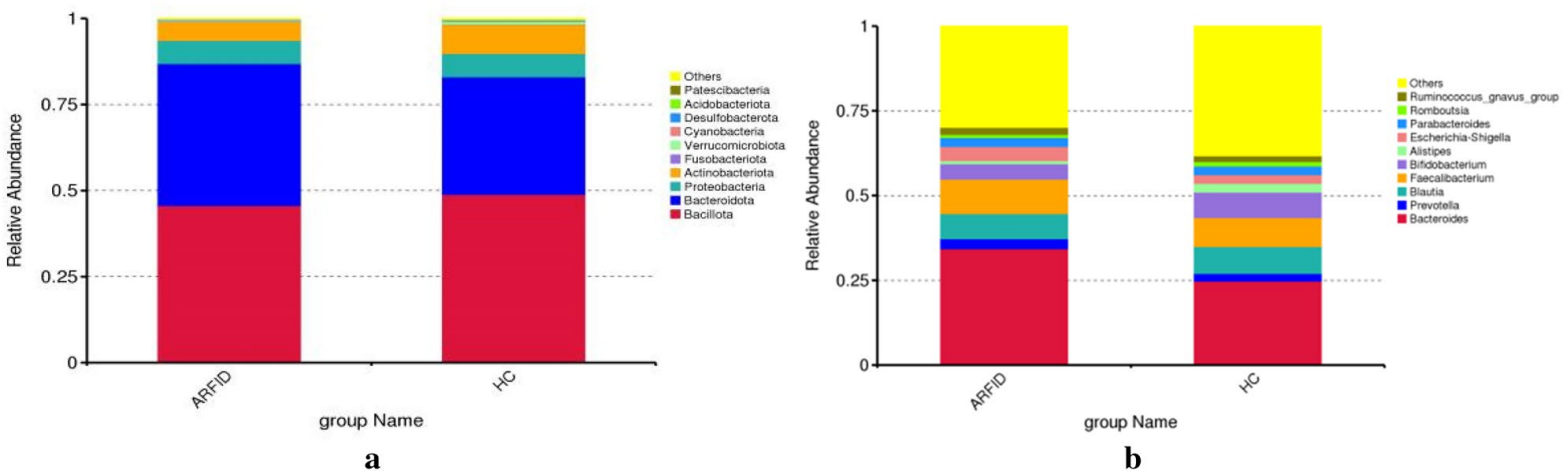
(**a**) The top 10 microbes at the phylum level. (**b**) The top 10 microbes at the genus level. The vertical axis is the relative abundance of each species. Columns with different colors correspond to different species, and the height of the column represents the abundance of the species.

At the phylum level, Bacillota were the most abundant bacteria in the ARFID group, followed by Bacteroidota, Proteobacteria and Actinobacteriota, which accounted for 45.7%, 41.2%, 6.8% and 5.4%, respectively. Similar to the ARFID group, Bacillota, Bacteroidota, Proteobacteria and Actinobacteriota were the most abundant bacteria in the HC group, accounting for 48.9%, 34.2%, 6.8% and 8.4%, respectively, but there were no statistically significant differences between the abundance of these microbial taxa in the two groups. At the genus level, the top 4 taxa in the ARFID group and HC group were Bacteroides, Faecalibacterium, Blautia and Bifidobacterium (Fig. 4). The abundance of Bacteroides was higher in the ARFID group than in the HC group, but the difference was not statistically significant (*P* > 0.05).

**Figure 5:**
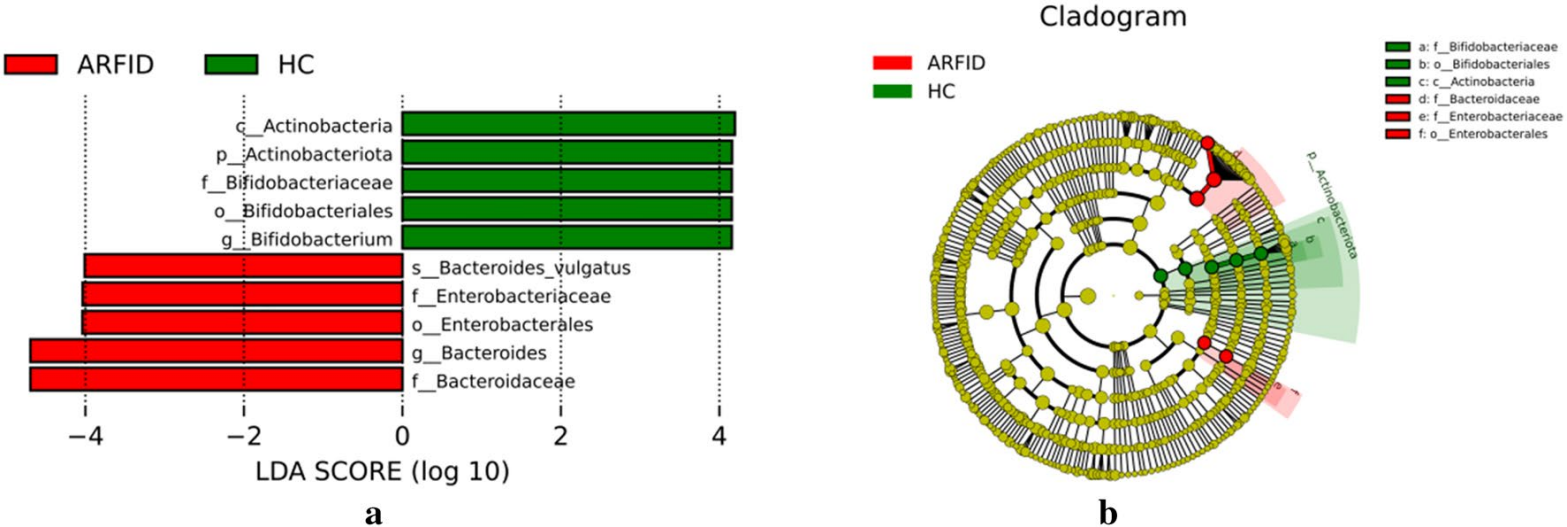
(**a**) Biomarkers associated with the ARFID group and HC group discovered by LEfSe analysis (logarithmic LDA score threshold = 4.0) in ARFID samples. (**b**) Cladogram representing the phylogenetic relationship of biomarkers associated with the ARFID group and HC group identified through linear discriminant effect size (LEfSe) analysis in samples.

To further explore all alterations in the gut microbiotas of the ARFID group and the HC group, we used LEfSe analysis (LDA effect size) to identify the key taxa responsible for the differences in the compositions of the fecal microbiotas between the two groups. The abundance of 5 taxa (the phylum *Actinobacteriota* and its corresponding class *Actinobacteria,* the order *Bifidobacteriales* and corresponding family *Bifidobacteriaceae* ,the genus *Bifidobacterium*), were enriched in the HC group. However, the order *Enterobacterales* and its corresponding family *Enterobacteriaceae,* the family *Bacteroidaceae* and corresponding genus *Bacteroides,* the species *Bacteroides vulgatus* were enriched in the ARFID group (Fig. 5).

### Composition and diversity of the microbiome in Y1 group and Y2 group determined by 16S rDNA sequencing

**Figure 6:**
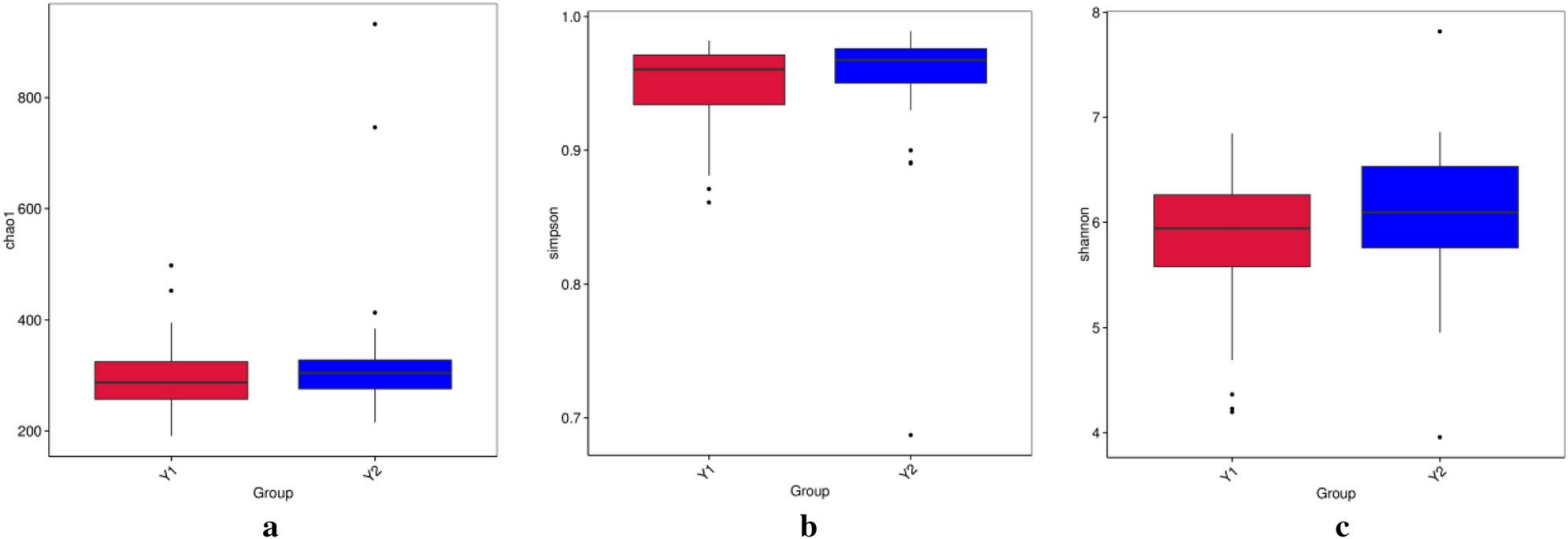
Comparison of the Chao1 index (**a**), the Simpson index (**b**) and the Shannon index (**c**) between the Y1 group and Y2 group.

To further verify the difference in the gut microbiota of children with ARFID at different ages, we divided the fecal samples of 102 children with ARFID into two groups according to age. Among them, the Y1 group contained a total of 59 children aged between 3 and 6 years, including 25 males and 24 females, and the Y2 group contained a total of 43 children aged between 7 and 12 years, including 12 males and 21 females. As shown in Fig. 6, there were no statistically significant differences between the Y1 group and Y2 group in the Chao1 index, Shannon index and Simpson index (*P* = 0.1, *P* = 0.06, *P* = 0.06).

**Figure 7:**
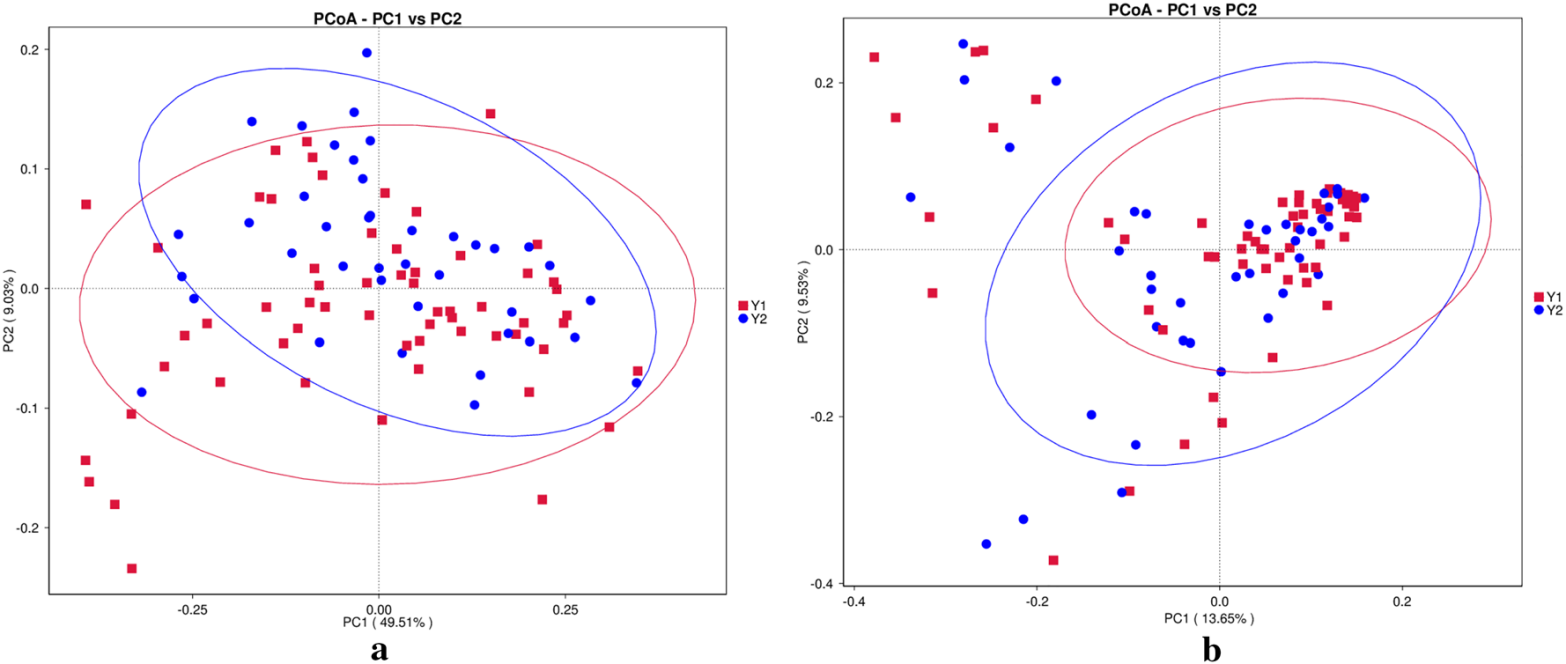
Principal coordinate analysis (PCoA) plots of individual fecal microbiotas based on weighted UniFrac (**a**) and unweighted UniFrac distances (**b**).

PCoA showed that the structural differences between the Y1 group and Y2 group were not significant (Fig. 7).

**Figure 8:**
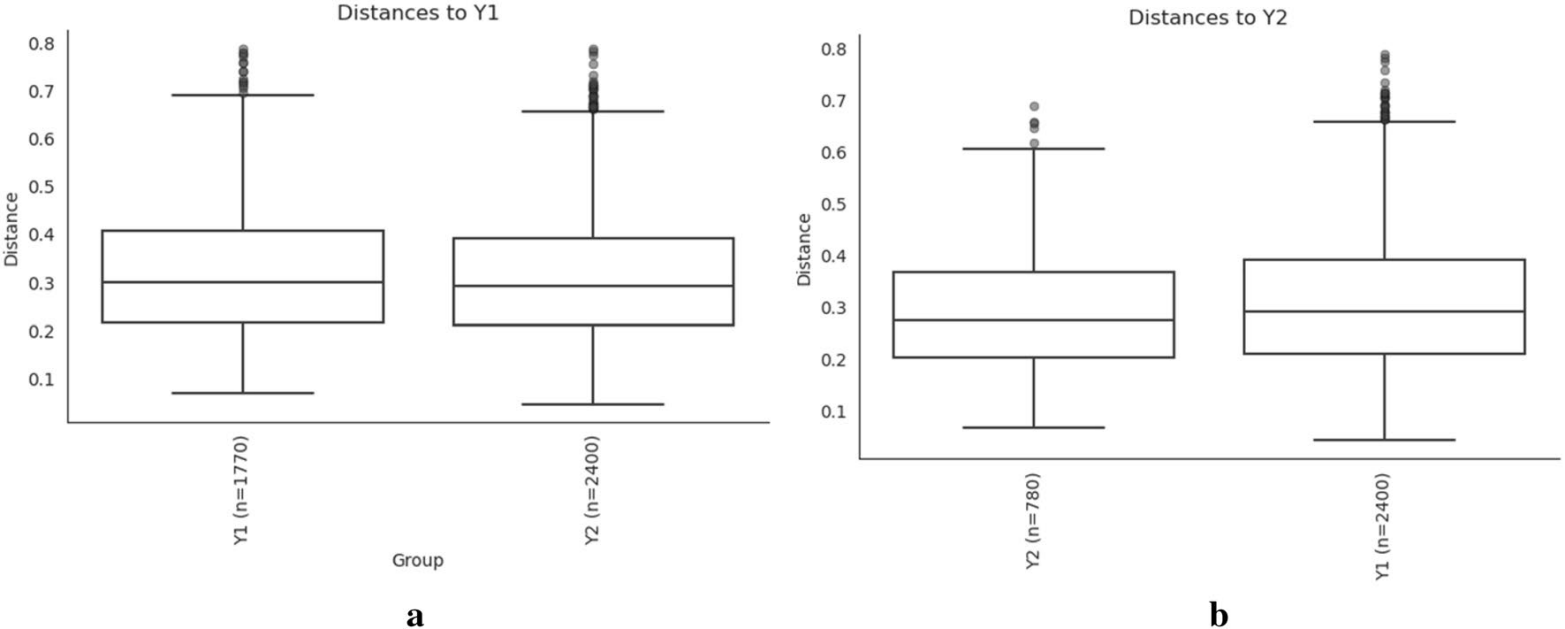
Anosim between the Y1 group and Y2 group. The y-coordinate is the distance, and the x-coordinate is the number of distances. R-values are between (− 1, 1), and R-values > 0 indicate differences between groups; R value < 0 indicates that the difference within the group is greater than the difference between the groups. *P* < 0.05 indicates statistical significance.

Moreover, Anosim showed no significant differences in bacterial community structures between the two groups (R-value = − 0.014, *P* = 0.7) (Fig. 8), so we concluded that there was no apparent difference in gut microbiota diversity between the Y1 group and Y2 group.

**Figure 9:**
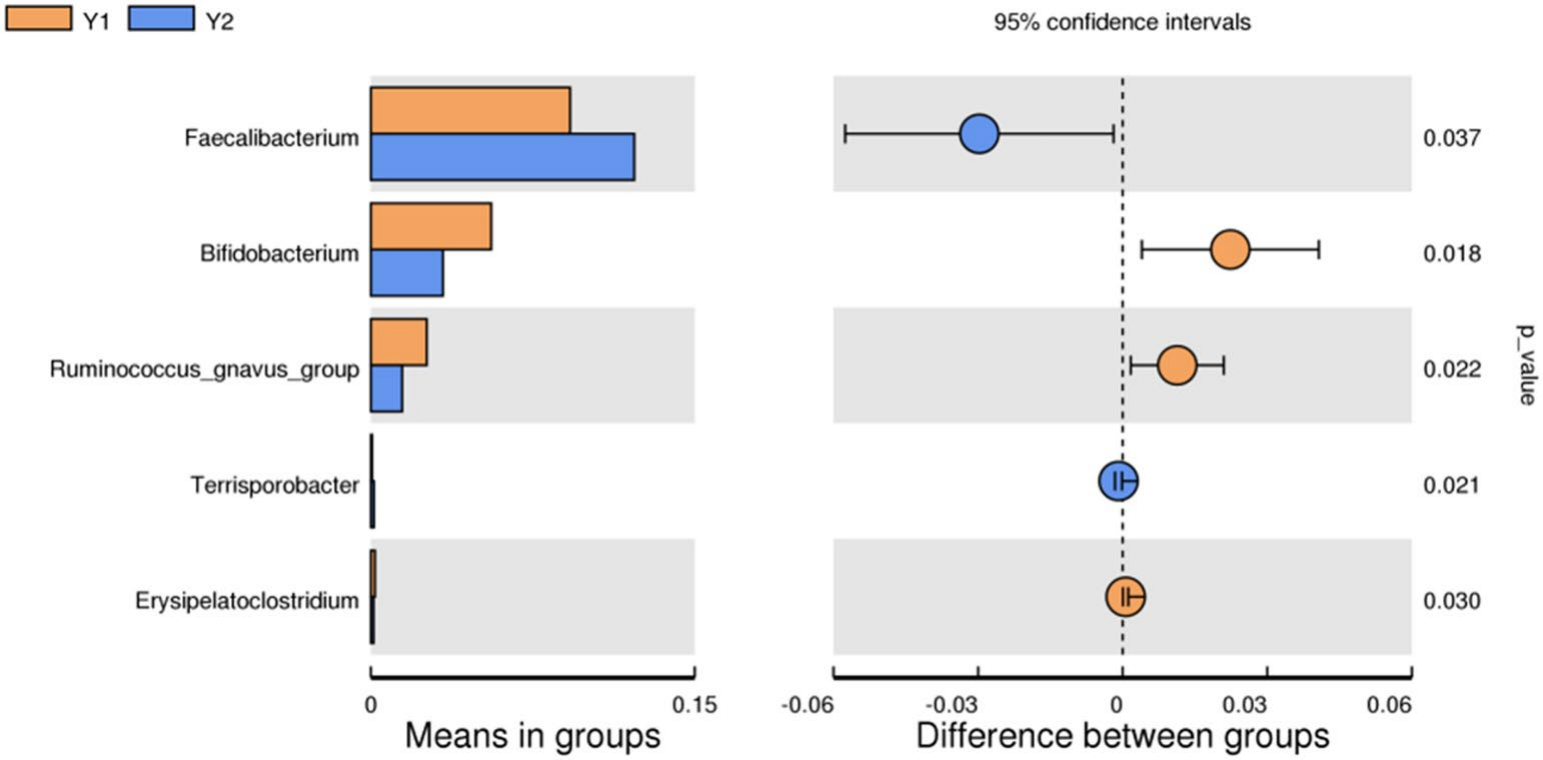
Differences between the Y1 group and Y2 group at the genus level as determined by T test analysis.

At the phylum level, Bacillota, Bacteroidota, Proteobacteria and Actinobacteria were the most abundant bacterial taxa in the two groups, and there were no statistically significant differences in the abundance of these taxa (*P* = 0.958, *P* = 0.456, *P* = 0.473, *P* = 0.065). Notably, at the genus level, the abundance of Faecalibacterium was higher in the Y2 group than in the Y1 group, and the difference was statistically significant (*P* = 0.037) (Fig. 9).

### Composition of the microbiome in the ARFID group and HC group as determined by metagenome sequencing

**Figure 10:**
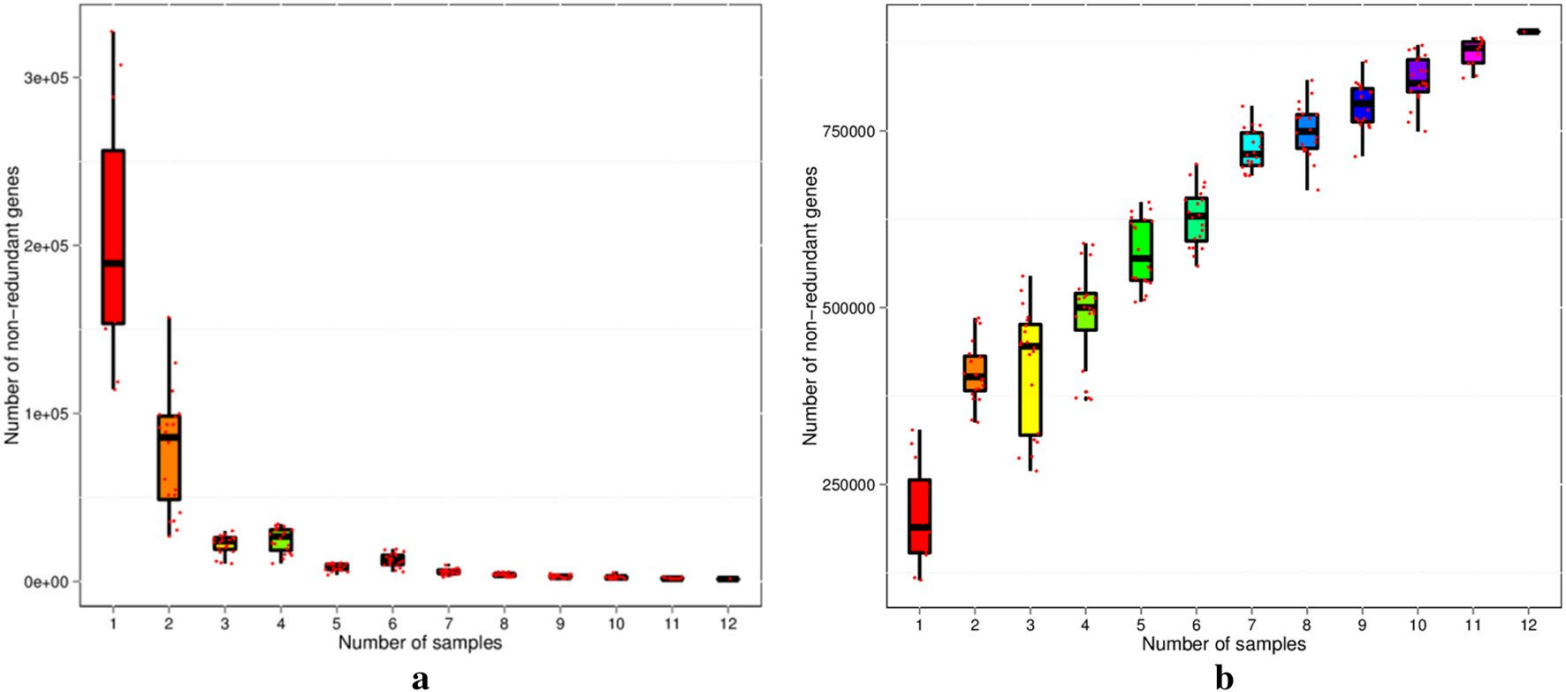
Rarefaction curves of the core genes (**a**) and pan genes (**b**).

We selected 12 samples through Distinct mode in the microPITA analysis for metagenome sequencing. After filtering and assembly, 952,862 unigenes were obtained, and the total length was 709.59 Mbp, with an average length of 744.7 bp. The dilution curve of the core genes (Fig. 10a) and pan genes (Fig. 10b) showed that the samples we measured could cover the real genes in the tested samples and meet the requirements of subsequent analyses.

**Figure 11:**
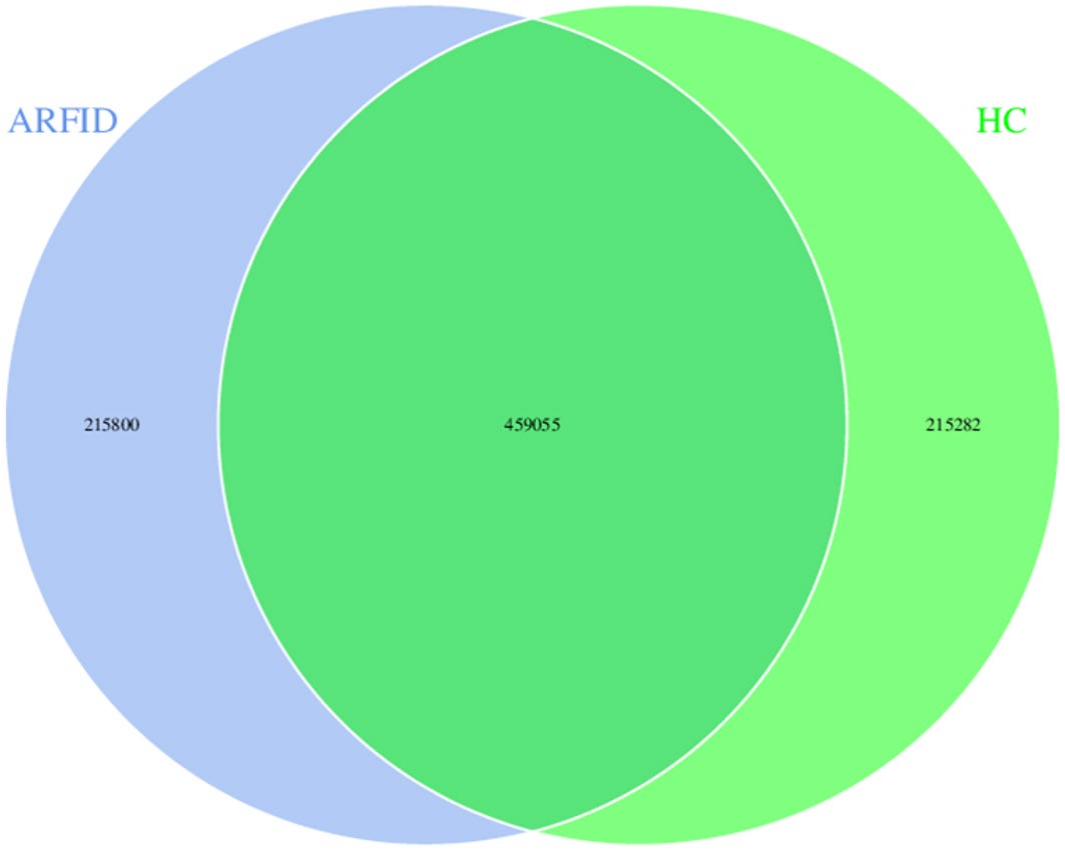
Venn diagram between the ARFID group and the HC group.

As shown in Fig. 11, there were 459,055 genes in common between the two groups, and the ARFID group had more gene entries, with 215,800 genes.

**Figure 12:**
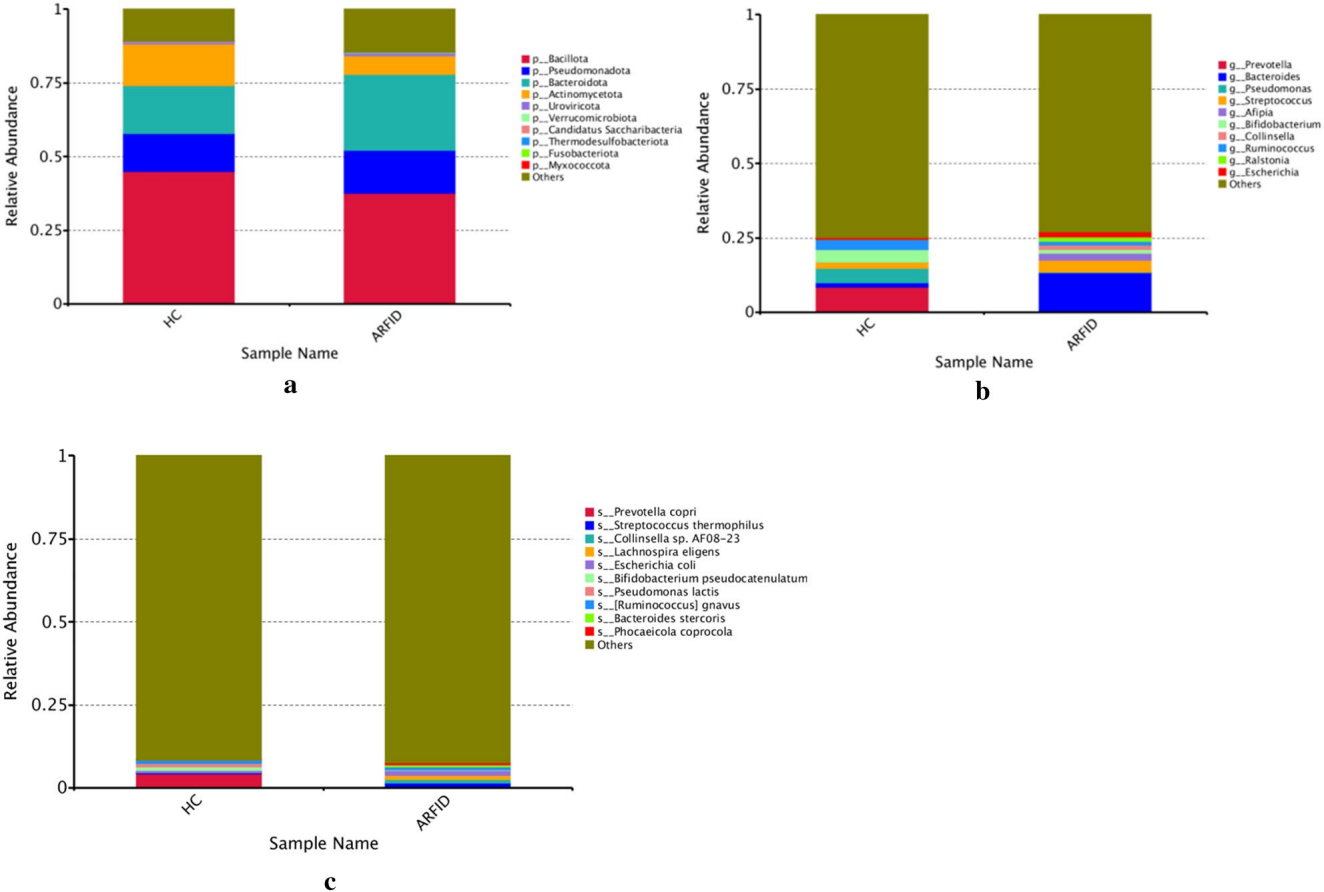
The top 10 microbes at the phylum level (**a**), genus level (**b**) and species level (**c**). The vertical axis is the relative abundance of each species. Columns with different colors correspond to different species, and the height of the column represents the abundance of the species.

The most abundant phyla in both groups were Bacillota, Bacteroidota, Pseudomonadota and Actinomycetota; the abundances of Bacillota and Actinomycetota in the HC group (45.1%, 14.3%) were higher than those in the ARFID group (37.6%, 6.3%), while the abundance of Bacteroidetes in the ARFID group (25.9%) was higher than that in the HC group (16.2%), although the difference was not statistically significant (*P* = 0.394) (Fig. 12a). At the genus level, different from the result of 16S rDNA sequencing, the abundance of Bacteroides in the ARFID group was much higher than that in the HC group (*P* = 0.041) (Fig. 12b); *Escherichia coli, Streptococcus thermophilus* and *Lachnospira eligens* were the most abundant taxa in the ARFID group at the species level, and *Prevotella copri*, *Bifidobacterium pseudocatenulatum*, and *Ruminococcus gnavus* were the top three microbial taxa in the HC group (Fig. 12c).

### Functional differences between the ARFID and HC groups

**Figure 13:**
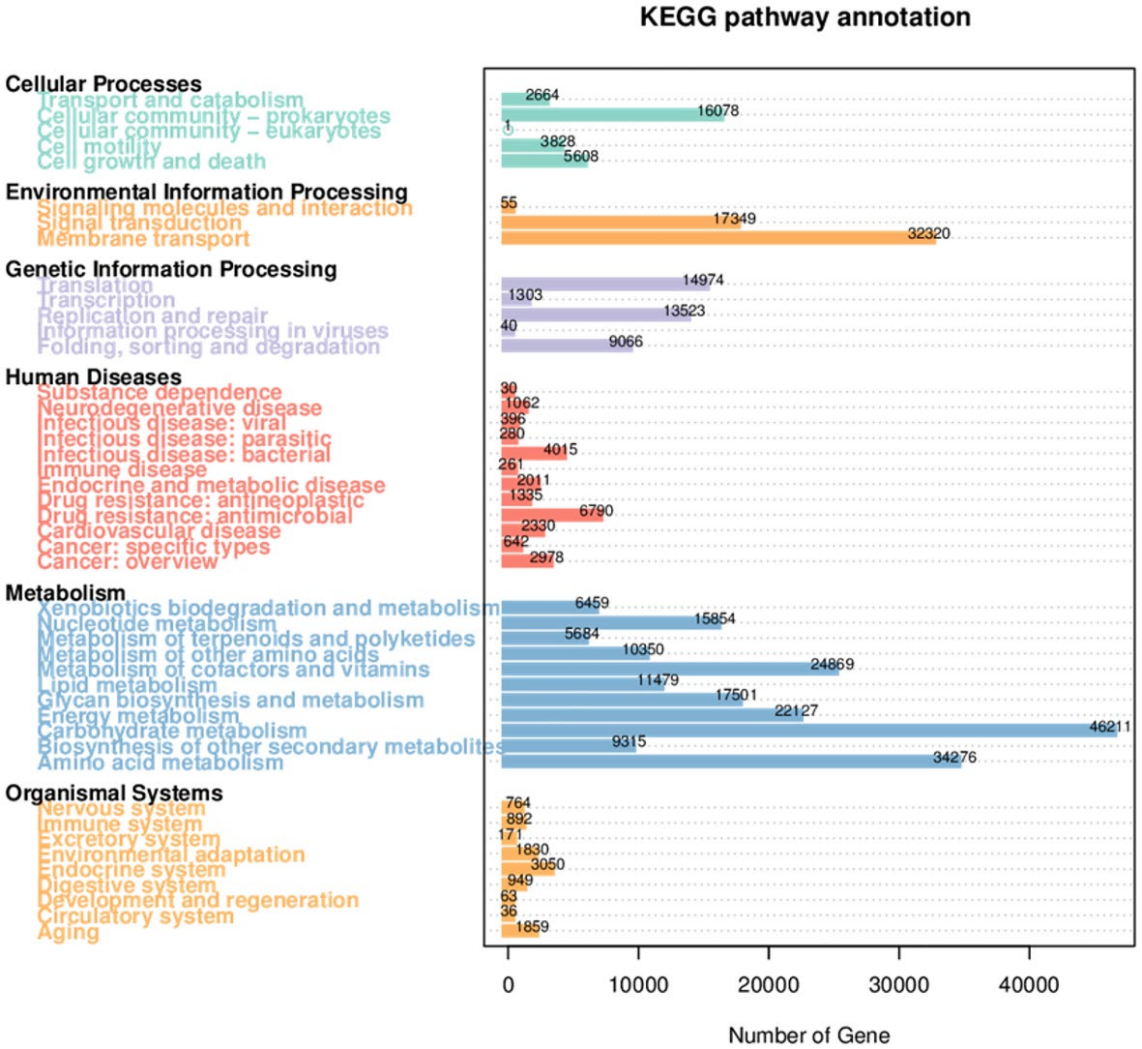
The number of genes in KEGG Level 1.

The KEGG database (Kyoto Encyclopedia of Genes and Genomes, Version: 2018.01) and the CAZy database (Carbohydrate-Active-enzymes Database, Version: 2018.01) are two commonly used databases. After blasting the filtered genes against the KEGG database, 6948 KOs were obtained. In KEGG Level 1, we found that a total of 44 pathways were related to the fecal microbiome, and the most abundant pathway was metabolism (Fig. 13).

In KEGG Level 2, carbohydrate metabolism, amino acid metabolism and environmental information processing: membrane transport were the most abundant pathways in the ARFID group and HC group (Table 2).

**Table 2:** The top 10 most abundant pathways in KEGG Level 2 (%).

Pathway	Metabolism: carbohydrate metabolism	Environmental information processing: membrane transport	Metabolism: amino acid metabolism	Metabolism: metabolism of cofactors and vitamins	Metabolism: energy metabolism	Environmental Information processing: signal transduction	Genetic information processing: translation	Cellular processes: cellular community-prokaryotes	Metabolism; glycan biosynthesis and metabolism	Metabolism; nucleotide metabolism
HC	4.568	3.219	3.485	2.422	2.282	1.654	2.062	1.583	1.523	1.664
ARFID	4.800	3.018	3.344	2.531	2.284	1.798	1.783	1.579	1.753	1.612
P value	0.589	0.937	0.485	0.485	1.00	0.180	0.240	0.937	0.180	0.485

In KEGG Level 3, ko02010 (ATP-binding cassette transporter) and ko02020 (Two-component system) were the most abundant pathways in both groups (Table 3), but the differences were not statistically significant (*P* > 0.05).

**Table 3:** The top 10 most abundant pathways in KEGG Level 3 (%).

Pathway	ko02010	ko02020	ko02024	ko03010	ko00230	ko00500	ko02060	ko00010	ko00520	ko00270
HC	2.265	1.324	1.028	1.252	1.015	0.885	0.325	0.683	0.804	0.728
ARFID	1.944	1.429	1.047	1.066	0.970	0.822	0.437	0.727	0.867	0.663
*P* value	0.394	0.240	0.937	0.310	0.485	0.818	0.937	0.699	0.240	0.180

**Figure 14:**
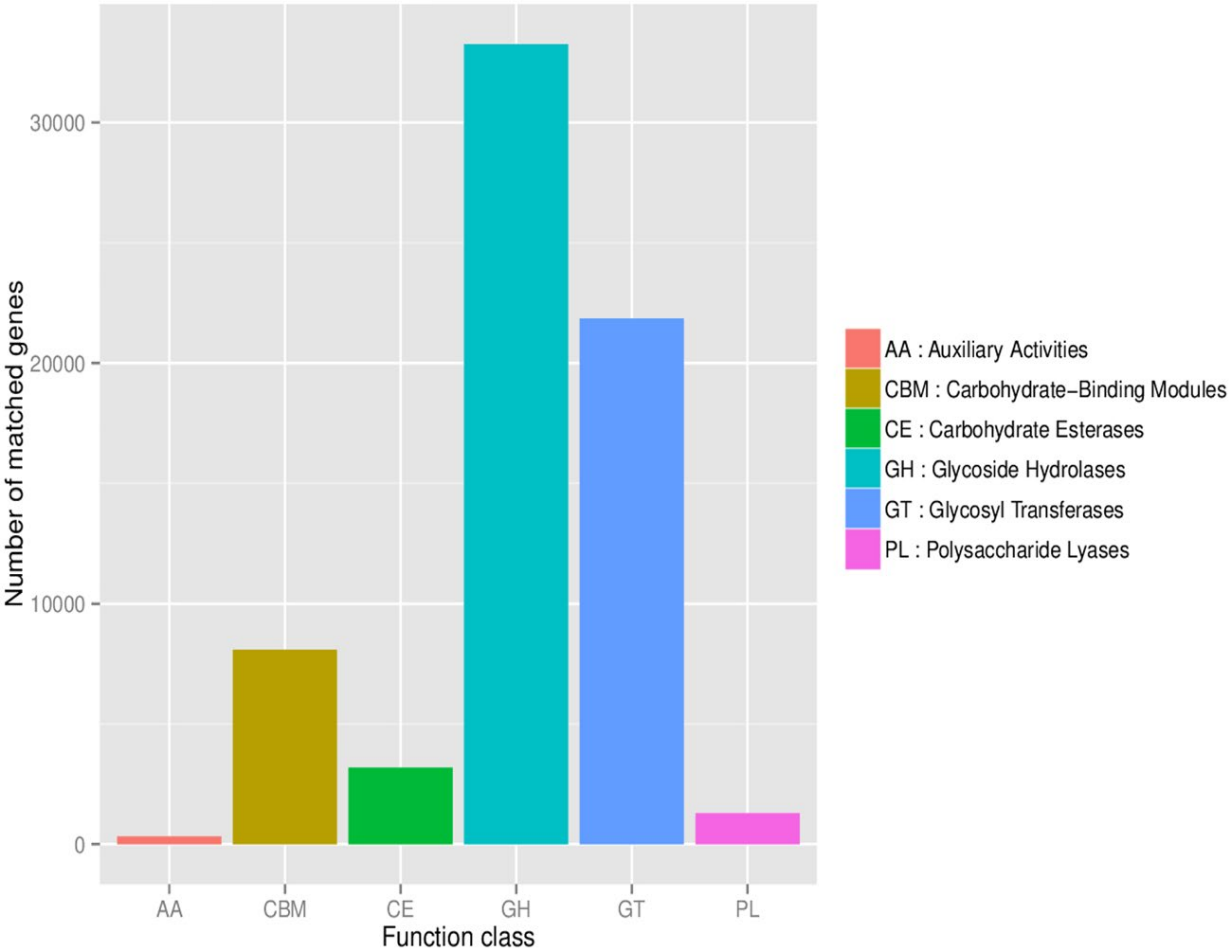
The number of CAZy annotations.

Based on the CAZy database, 541 ECs were identified. Six major enzymes were found in the gut microbiota: glycoside hydrolase (GH), glycosyl transferases (GT), carbohydrate binding module (CBM), carbohydrate esterase (CE), polysaccharide lyase (PL) and auxiliary oxidoreductase (AA). Glycoside hydrolase was the most annotated gene in the two groups (Fig. 14).

**Figure 15:**
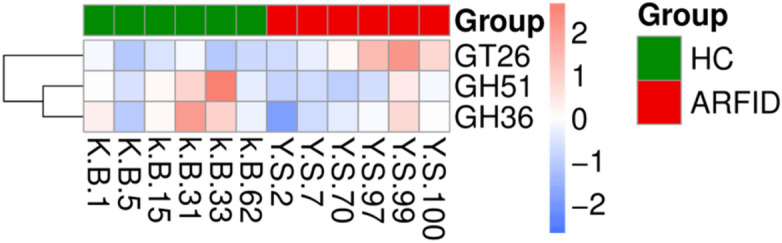
Cluster plot of the abundance of functions in Level 2 between groups based on the CAZy annotations.

As shown by the clustering heatmap, GT26 was significantly more abundant in the ARFID group, while GH51 and GH36 were more abundant in the HC group (Fig. 15).

**Figure 16:**
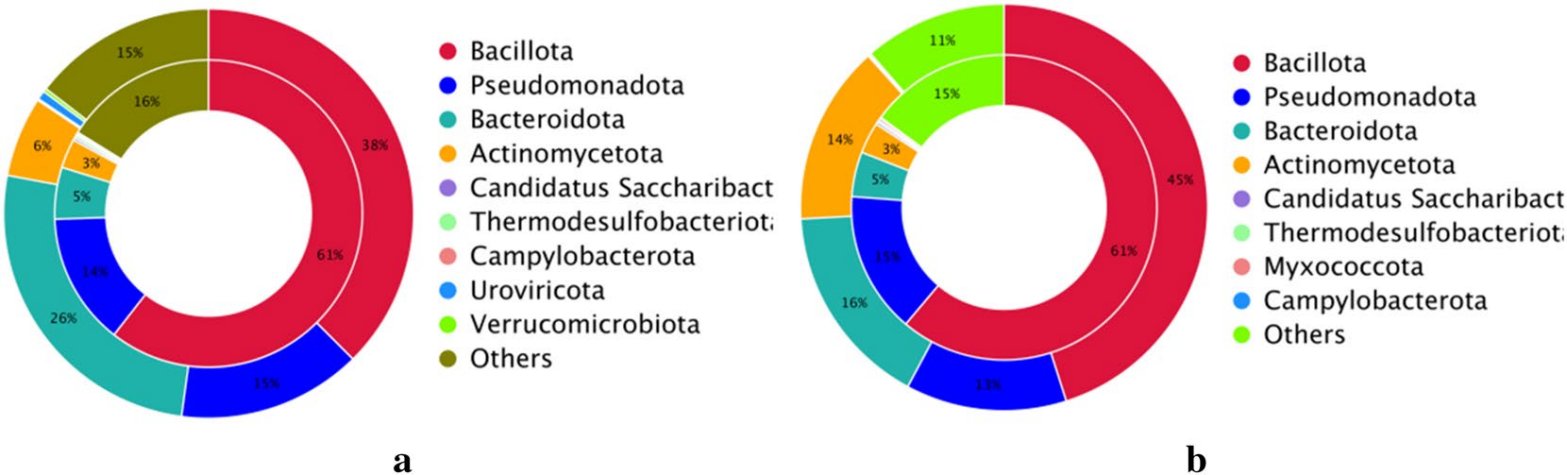
Double-circle diagram of the relationship between two groups of drug resistance genes and species attribution. (**a**) is the ARFID group, (**b**) is the HC group, the inner circle is the species distribution of AROs, and the outer circle is the species distribution of all sample genes.

Based on the CARD database (v2.0.1), we found a total of 195 resistance genes. It can be seen from the species attribution diagram of resistance genes that Bacillota, with the highest content of resistance genes in the both groups, accounted for 38% of all drug-resistant bacteria in the ARFID group and 45% in the HC group (Fig. 16).

**Figure 17:**
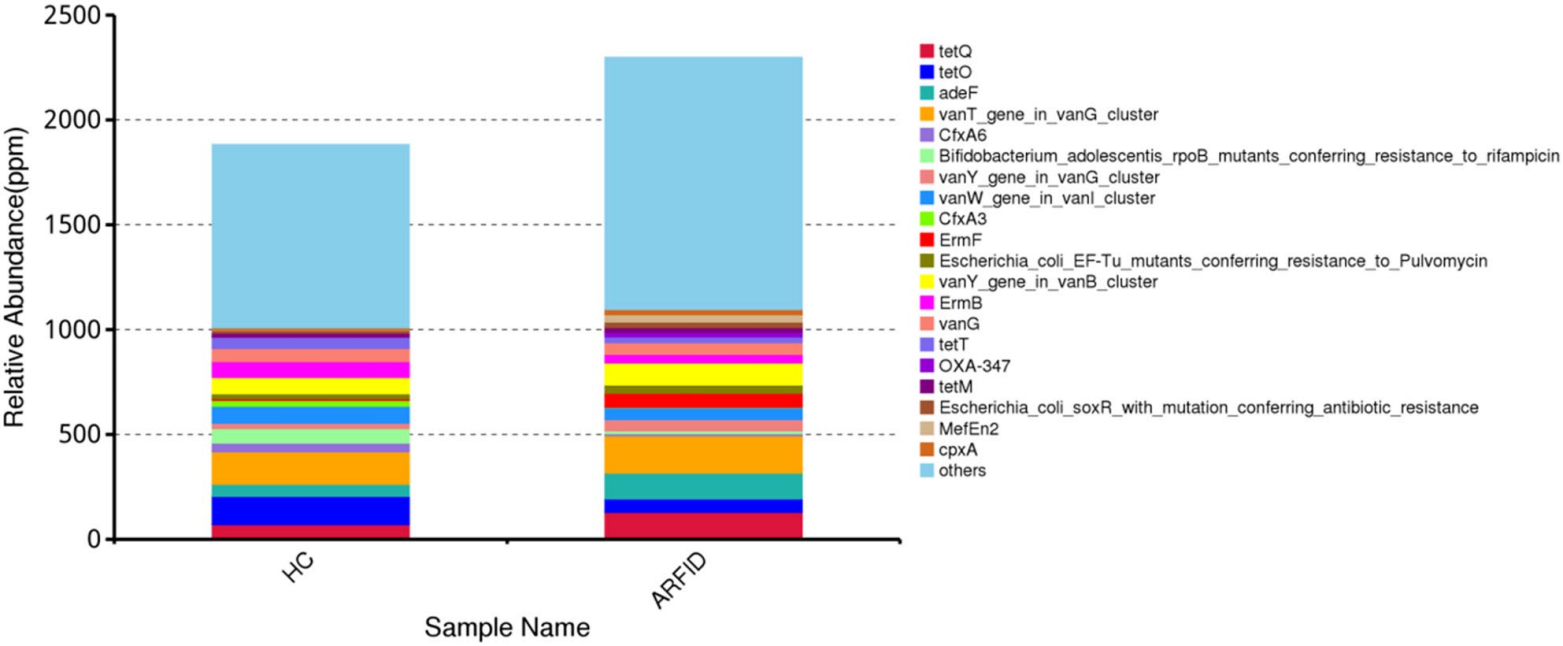
Abundance of antibiotic resistance (AR) genes in the ARFID group and HC group.

The most abundant antibiotic resistance genes in the ARFID group were vanT (vancomycin resistance gene), followed by tetQ (tetracycline resistance gene), adeF (*Acinetobacter baumannii* resistance gene), and ermF (macrolide resistance gene), while vanT, tetO, vanW (vancomycin resistance gene) and vanY (vancomycin resistance gene) were the 4 most abundant antibiotic resistance genes in the HC group (Fig. 17). The abundance of ermF in the ARFID group was significantly higher than that in the HC group (*P* = 0.041).

## Discussion

The human body carries a large number of gut microbes, which affect our metabolism, hormonal status, immune system and even behavior^25^, therefore, the human microbiome is becoming an important research topic in somatic and psychiatric diseases^26, 27^. Though research into ARFID is increasing^28^, none research on the gut microbiota of children with AFRID have been found so far. Recently, the most widely used high-throughput sequencing technique for bacterial identification has been 16S rDNA sequencing^29^, but bacteria can only be identified to the genus level, and specific information at the species level cannot be obtained. However, compared with 16S rDNA sequencing, metagenome sequencing can not only solve this problem but also obtain functional information on the microbiome. Based on 16S rDNA sequencing and metagenome sequencing, this study analyzed the composition of the gut microbiota in children with ARFID and healthy children and found that there were differences in the structure, diversity and functional information of the gut microbiome between the two groups.

In our study, the Chao1index (reflecting the microbe species richness), was higher in the healthy children than in the children with ARFID, and the difference was statistically significant (*P* < 0.001). And the Shannon index and Simpson index (reflecting microbial species diversity) in the ARFID group, were higher than that in the HC group, and the differences were statistically significant (*P* = 0.009 and *P* = 0.02). These results suggesting that intestinal flora community structure and diversity changed compared with those in healthy children and that intestinal flora disorders occurred in children with ARFID. Due to the different databases that used in the process of metagenome and 16S rDNA sequencing, as well as the different depth of sequencing, there will be differences in the annotation results. For example, the most abundant microbes in the two groups at the phylum level were Bacillota, Bacteroidota, Proteobacteria and Actinobacteriota through 16S rDNA sequencing, however, the most abundant phyla in both groups were Bacillota, Bacteroidota, Pseudomonadota and Actinomycetota at the phylum level through metagenome sequencing. Besides, At the genus level, different from the result of 16S rDNA sequencing, the abundance of Bacteroides in the ARFID group was much higher than that in the HC group (*P* = 0.041), in the result of metagenome sequencing. In order to further understand the differential abundance at the species level between the two groups, we conducted metagenomic analysis of some samples. However, there were no statistically significant differences between the abundance of these microbial taxa in the two groups at the species level. Based on the LEfSe analysis, the microbes that had a strong influence in the ARFID group were the order *Enterobacterales* and its corresponding family *Enterobacteriaceae,* the family *Bacteroidaceae* and corresponding genus *Bacteroides,* the species *Bacteroides vulgatus,* while the phylum *Actinobacteriota* and its corresponding class *Actinobacteria,* the order *Bifidobacteriales* and its corresponding family *Bifidobacteriaceae*, the genus *Bifidobacterium* influenced the HC group most.

Previous studies revealed that Bacteroides is a dominant genus in human intestines^30^. They can colonize the host intestines for a long time and have the function of regulating the intestinal microenvironment^31^, using carbon hydrate, enhancing the host’s adaptability to the environment^32, 33^ and secreting metabolites such as short-chain fatty acids (SCFAs)^34, 35^, thus establishing a stable symbiotic relationship with the host^36^. However, some reports have also shown that Bacteroides have adverse effects on the host^37–39^. Bacteroides vulgaris is widely considered a class of Bacteroides related to inflammatory bowel disease. This conjecture has been verified in a large number of in vivo and in vitro experiments^40–43^. In addition, some animal experiments have proven that *Bacteroides vulgaris* alleviates inflammation in mice^44, 45^. The family Enterobacteriaceae has had a great medical and public health impact on the global community, as these species are associated with a wide range of clinical syndromes and are major causative agents of foodborne enteritis^46^. So far, a large number of studies have focused on the epidemiology, pathogenesis, virulence, and/or antibiotic resistance of pathogenic strains of Enterobacteriaceae in humans^47^. Bifidobacterium, belonging to Actinobacteria, is a genus of gram-positive, pleomorphic, rod-shaped bacteria that are strictly anaerobic, and these bacteria have important immune regulation, anti-tumor, anti-pathogenic, anti-inflammation, anti-aging, and hypolipemic effects in humans^48^. Therefore, Bifidobacterium species have long been used as probiotics to alleviate various diseases by changing the gut microbiota composition and are significantly associated with human health^49^. The results of our study suggested that the increased content of Bacteroides or Enterobacteria or the reduced content of Bifidobacterium may be related to ARFID, but the pathogenesis still needs further study. As is known to all, a persistent failure to meet appropriate nutritional is one of the main characteristics of ARFID^50, 51^. Growing evidence indicates that malnutrition may bring about qualitative changes in the microbiome^52^ and specific strains of probiotics can potentially address the qualitative shift that occurs in the malnourished microbiome^53^. Previous study have shown that the depletion in gut Bifidobacterium represents the first step in gut microbiota alteration that associated with severe malnutrition^54^, suggesting that Bifidobacterium supplementation may be extremely important in the treatment of malnutrition. Although the best therapeutic intervention for eating disorders (including ARFID) is currently family-based intervention, in conjunction with both medical and dietetic monitoring and management, and pharmacologic management is never recommended as a first-line treatment, probiotic preparations may be useful as adjunctive interventions, similar to the previous researches, the live Bifidobacterium preparation may be a good choice through our study.

The composition and function of the gut microbiota have been largely overlooked in 3–6-year-old and 6–12-year-old children compared with infants and adolescents^55^, probably because such investigations have been constrained by ethical and practical considerations, such as difficulties in obtaining fecal samples from individuals in these age groups^56^. In our results, there seemed to be no difference between preschool children and primary school children with ARFID in terms of gut microbiota diversity. At the phylum level, the most abundant bacteria in the two groups were the same; however, at the genus level, the abundance of Faecalibacterium was significantly higher in primary school children than in preschool children with ARFID, while the abundance of Bifidobacterium and *Ruminococcus gnavus* were higher in preschool children with ARFID. Faecalibacterium is a genus of strictly anaerobic, extremely oxygen-sensitive (EOS), Gram-positive bacteria and is considered to be ubiquitous in the gastrointestinal tracts of healthy humans^57^. Numerous types of evidence suggest that Faecalibacterium plays an important role in immune system regulation, gut barrier protection, and microbiota modulation^58^. Notably, due to Faecalibacterium increased with age from newborns to adults and decreased again at later ages^59^, the Faecalibacterium was more abundant at the genus level in older children with ARFID in our study, which may related to age gain, however, more researches are needed to support this hypothesis.

Finally, according to the KEGG annotation results, there seemed to be no significant difference in gut microbiota function between children with ARFID and healthy children. However, GT26 was significantly enriched in children with ARFID compared with healthy children in Level 2 based on the CAZy database. It was reported that based on the structural relatedness of glycosyltransferase (GT) catalytic and carbohydrate-binding modules, GTs have been grouped into 115 families in the CAZy database, and only four distinct GT protein folds, termed GT-A through GT-D, have been reported thus far^60^. The architecture of GT-B enzymes consists of two β/α/β Rossmann-like domains, and the GT26 family was predicted to possess the GT-B fold^61^, but there have been no other further reports on GT26 to date.

The human gut microbiota is an important reservoir of antibiotic resistance genes (ARGs). Due to improper use of antibiotics, the gut microbiota will be disordered, and the presence of drug-resistant bacteria can affect the intestinal microecological environment, which may accelerate the development of disease. A recent report suggested that tetracycline, multidrug, and macrolide-lincosamide streptogramin (MLS) resistance genes were the top three most abundant ARG types in healthy individuals, and ermF was a representative ARG in the Chinese population^62^. Unlike findings in adults, our results suggested that the most abundant genes for gut microbiota resistance in healthy children were vancomycin, tetracycline and macrolide resistance genes. ErmF is a subtype of the MLS resistance gene type. In our study, we found that the ermF level was significantly higher in children with ARFID, which may be due to the higher abundance of Bacteroides. Since the use of macrolide antibiotics is the main reason for the increase in the abundance of Bacteroides^63^, we should be more cautious in the use of macrolide antibiotics in clinical practice. At present, we still know little about the impact of resistance genes on the gut microbiota in children, and there is not enough evidence to prove that resistance genes are directly related to the occurrence and development of ARFID, but researchers cannot ignore the potential threat of resistance genes to children’s health.

This was a preliminary study of the intestinal flora in children with ARFID, however, some limitations were identified. First, because of the high cost of metagenome sequencing, the total sample size was relatively limited. Moreover, due to the lack of healthy volunteers, the number of healthy controls was small. Finally, the sampling area was limited. To overcome these limitations, future studies should control related confounding factors and apply multiomics research strategies to microbial studies of ARFID, which will help reach more complete and in-depth conclusions.>

## Data Availability

The raw sequence data reported in this paper have been deposited in the Genome Sequence Archive (Genomics, Proteomics & Bioinformatics 2021) in National Genomics Data Center (Nucleic Acids Res 2022), China National Center for Bioinformation / Beijing Institute of Genomics, Chinese Academy of Sciences (GSA-Human: HRA005177) that are publicly accessible at https://ngdc.cncb.ac.cn/gsa-human.

## Abbreviations

AA: Auxiliary oxidoreductase
AN: Anorexia nervosa
ARGs: Reservoir of antibiotic resistance genes
ARFID: Avoidant/restrictive food intake disorder
BED: Binge eating disorder
CBM: Carbohydrate binding module
CE: Carbohydrate esterase
DSM-5: Diagnostic and statistical manual of mental disorders
EOS: Extremely oxygen-sensitive
FEDs: Feeding and eating difficulties
GH: Glycoside hydrolase
GT: Glycosyltransferase
ICD-11: The 11th revision of the World Health Organization’s international statistical classification of diseases
MLS: Macrolide-lincosamide streptogramin
PL: Polysaccharide lyase
RD: Rumination disorder
SCFAs: Short-chain fatty acids
